# The Influence of Cat Coat Colour, Eye Shape, and Pupil Size on Ratings of Adoptability Based on a Standardised Online Image, in an Australian Sample

**DOI:** 10.3390/ani16020339

**Published:** 2026-01-22

**Authors:** Sarah C. Whelan, Deanna L. Tepper, Jessica K. Dawson, Diana Rayment, Lily Chilcott, Pauleen C. Bennett, Tiffani J. Howell

**Affiliations:** 1 School of Psychology and Public Health, La Trobe University, Bendigo, VIC 3552, Australia; 21232335@students.latrobe.edu.au (S.C.W.); d.tepper@latrobe.edu.au (D.L.T.); jessica.dawson@latrobe.edu.au (J.K.D.); 20940254@students.latrobe.edu.au (L.C.); 2 PetRescue, Perth, WA 6000, Australia; diana.rayment@petrescue.org.au

**Keywords:** pet rescue, cat sociability, cat cuteness, black cat bias

## Abstract

Online pet adoption profiles, which present images of available pets, can create powerful first impressions. Research about factors influencing pet adoptability online is limited and has mainly focused on dogs, yet cats are also in need of homes. People may judge a cat’s behaviour based on physical factors, like coat colour, eye shape, and pupil size. This study aimed to determine whether those physical traits relate to cat adoptability and, if so, whether they might do this through influencing perceptions of cuteness and specific behaviours. Australian adults (*N* = 624) were surveyed online using a single cat image that was modified to have all possible combinations of four different coat colours, three eye shapes, and three pupil sizes. Adoptability scores varied significantly with the cat’s coat colour and eye shape, but not pupil size. Black cats with walnut-shaped eyes had the highest adoptability ratings. A stepwise multiple regression determined seven variables influencing adoptability scores, including the degree to which the image was rated as cute, friendly, shy, active, difficult, playful, and devoted. Cuteness had the strongest influence, explaining 34% of the model variance. Multiple two-way ANOVAs compared these seven variables across combinations of coat colour and eye shape, with black cats being perceived as significantly more friendly and playful. This information may help animal adoption agencies understand how cats’ physical features can be utilised in online profiles to enhance adoption outcomes.

## Simple Summary

Online cat adoption profiles let people see available cats before visiting a shelter or rescue facility. People may choose to adopt a cat based on physical characteristics or temperament traits like friendliness, but it is unclear whether these characteristics are obvious in profile photos. The aim of this study was to measure the impact of physical traits on adoptability ratings and to determine whether these traits relate to perceptions of cuteness and cat personality. We manipulated a single cat image to create 36 images with different combinations of coat colour, eye shape, and pupil size. We surveyed 624 Australian adults, each of whom looked at one image, rating adoptability, cuteness, and traits like friendliness, shyness, and playfulness. Cats with black coats had high adoptability ratings, and the black cat with walnut-shaped eyes had the highest adoptability rating. Pupil size did not have an impact. Adoptability was also influenced by whether the cat was perceived as cute, friendly, and shy, with cuteness being the strongest contributor to adoptability ratings. Black cats had the highest ratings for desirable traits, like cuteness and friendliness. Shelters and rescues can use this information to ensure that profile photos reflect desirable cat traits.

## 1. Introduction

Online profiles have improved companion animal adoption rates by helping connect thousands of pets with potential owners [1], making them an important part of the advertising process [2]. However, visual appearances may not accurately depict an animal’s behavioural tendencies, which may be of greater importance in determining successful adoption than the animal’s appearance [3,4]. Hence, animal lives and consumer satisfaction depend on adoption agencies’ understanding of which aspects of images affect perceived adoptability.

Research in dogs has revealed that online profiles that present a non-blurry image of the dog standing up, maintaining direct eye contact with the camera, and captured with an outdoor location in the background are viewed more frequently and lead to higher adoption interest [2,3]. This suggests that contextual features are used by potential adopters to inform their choice. Depending on the breed of dog, however, images that displayed a dog with floppy ears compared to a dog with both ears erect had faster adoption rates [5], confirming that physical attributes are also influential. There is limited research investigating features of cat images that influence adoption rates, although online profiles that present a third-person description and describe the cat in a positive way are reported to be more effective [3,6,7]. Isgate and Couchman [8] also found that the presence of a toy, whether the cat was playing with it or not, increased the speed of adoption, perhaps because it conveys sociability. This suggests that, like dogs, contextual features are important in cat adoption.

Whether the physical features of cats are equally important is not known, but they are believed to likely influence perceptions of adoptability [8]. Some research suggests that coat colour is more important in the adoption of adult cats than kittens [9], but one study found that black cats and black kittens both took longer to be adopted than other colours [10]. In another study, light-coloured cats were adopted faster than dark-coloured cats, but the difference was not significant [11], and in a study from Sydney, Australia, white cats had longer lengths of stay than black cats [12]. There is also evidence that a cat’s coat colour predicts perceptions of their personality and behaviour, [13,14], but not necessarily ratings of adoptability [14]. There is some evidence that this bias may relate to superstitiousness [14] or belief in witchcraft among people who identify as dog people [15]. Although it is unclear to what extent these perceptions have any factual foundations, it is important to determine if individuals stereotype cats based on coat colour, as this information could inform strategies to ameliorate this effect when marketing cats that display desirable behaviours inconsistent with any stereotypes that exist.

Facial morphology is a feature of a cat’s appearance that may influence perceptions of adoptability. Baby schema refers to a set of physical features in human infants and children, often considered cute, that elicit parental nurturance and caregiving behaviours [16,17]. The eye region is critical for humans when scanning human faces, likely because this region reveals emotional and mental states [18], and this appears to apply to cats as well. A study by Jack and Carroll [6] explored whether eye shape was a reason for individuals finding some felines cuter than others, highlighting that the rounder the eye, the cuter the image was perceived to be. Another study has shown that eyes are significant in influencing bond formation between humans and pets, with cat–human bonds reported to be very similar to mother-infant and dog–human bonds [19]. It is believed that photos that capture a cat’s eyes and expression can instantly create a connection with the viewer [20]. However, it is not known if characteristics such as eye shape and pupil size can influence ratings of adoptability, cuteness, or behavioural traits like sociability.

In dogs, coat colour and eye characteristics interact to create a contested phenomenon called ‘black dog syndrome,’ which describes the universal under-adoption of black dogs in shelters [21]. This may be due to the coat colour affecting the visibility of the eye region, which reduces the overall ability for potential adopters to perceive the dog’s emotions [22]. It is not known if cats with coats that contrast poorly against their eyes are similarly perceived as less attractive or less adoptable. Eye shape or pupil size may have more impact in cats, where visibility of the eye is easier, regardless of other features.

In summary, research has identified limited ways to improve online cat adoptions, which have become an increasingly important avenue for finding new homes for thousands of homeless animals each year. Images are instrumental in driving adoption interest, but exactly which features of the image are important remains unknown. Context appears critical, but physical features of the animal may also be important. While these cannot be altered, knowing which ones are important and whether they interact with each other could allow adoption agencies to ameliorate any impact. Hence, the aim of this study was to explore whether digitally manipulating the eye shape, pupil size, and/or coat colour of a single cat image influenced perceptions of the cat’s adoptability and, if so, whether this may be due to effects on ratings of cuteness and perceived behavioural traits.

## 2. Materials and Methods

### 2.1. Participants

Prerequisites for participation in this study included being a current Australian resident, aged 18 years and over, and being able to comprehend English fluently. Participants were not required to be current or previous cat owners; however, it was anticipated that recruitment via PetRescue, a national animal welfare charity and pet adoption organisation, would ensure that the sample was heavily biased towards those with an interest in cat adoption. Overall, 864 participants commenced the survey. After removal of incomplete responses, a final sample of 624 remained.

### 2.2. Measures/Materials

This quantitative study utilised an online questionnaire conducted as part of a larger project that investigated other factors related to cat adoption. Only parts of the survey that are relevant to the aims of the current study are presented here. Participants were initially asked a series of demographic questions (e.g., year they were born) and about their knowledge and attitudes towards pet cats. Then, through randomization, they were presented with one of thirty-six images of a domestic shorthair cat (see Supplementary Materials for all photos).

The original photograph depicted a cat’s head and upper body, with green eyes (a common colour for domestic shorthair cats [23]), a neutral face, eyes facing forward and level with the camera, ears facing forward, and mouth closed, with no visible injuries or deformities being evident. Each participant was presented with the same image, which was manipulated using image-editing software, namely Adobe Photoshop 2020 version and AI ChatGPT tool 4o Image Generation (2024), so that 36 combinations of eye shape (one of round, walnut, and almond), pupil size (one of large, medium, and small), and coat-colour (one of black, tabby, ginger, and white) were included, with all possible combinations being covered. Eye shapes and coat colours were manipulated by taking the original image and utilising prompts via the AI tool until satisfactory results were obtained (see Supplementary Materials for prompts). Further refinements to the coat colour and eye shape were adjusted in Photoshop. The three pupil sizes were manipulated through Photoshop and were based on a paper by Espinheira Gomes et al. [24], which provided a sizing guide for cat pupils; in a life-size rendition of the image, the large pupil was 11 ± 1.5 mm, the medium pupil 5.1 ± 1.7 mm, and the small pupil 3.7 ± 2.3 mm.

**Figure 1:**
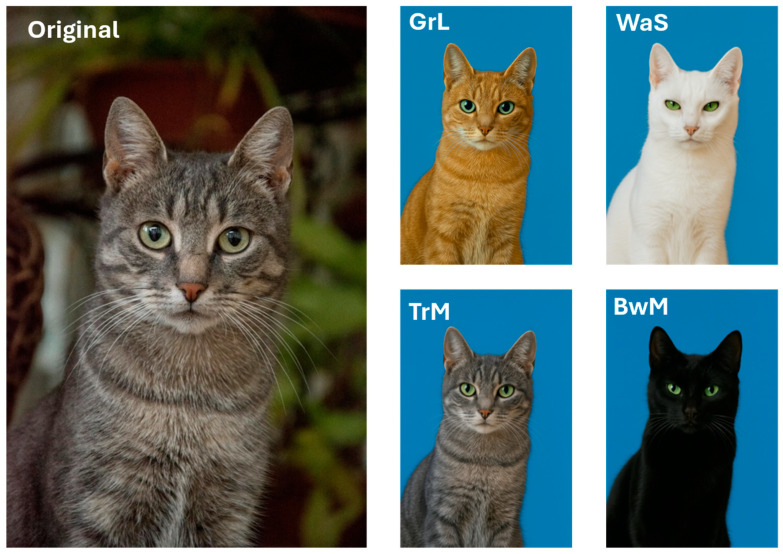
Example of original photograph compared to manipulated images. GrL = ginger-tabby cat with round eyes and large pupils. WaS = white cat with almond-shaped eyes and small pupils. TrM = grey tabby with round eyes and medium-sized pupils. BwM = black cat with walnut-shaped eyes and medium-sized pupils. The text presented on the images (e.g., ‘GrL’) was not visible on the survey images. Original by Claudia [27], CC BY 3.0. See Supplementary Materials for all 36 manipulated images.

The manipulated images all included the same solid blue background (see Figure 1), with a solid background colour being shown previously to keep focus solely on the animal being photographed [25]. The colour blue was chosen to contrast effectively against all chosen coat colours [20,26].

While viewing the image, which was repeated on each relevant page of the survey, participants were asked a series of 24 questions about the cat’s perceived cuteness, attractiveness, behavioural characteristics, and overall adoptability relative to other cats. This included questions based on 10 personality characteristics of cats identified by Delgado et al. [28] (*active*, *aloof*, *bold*, *calm*, *friendly*, *intolerant*, *shy*, *stubborn*, *tolerant*, *and trainable*), as well as additional questions generated by our research group (e.g., *how cute is this cat* and *how devoted to its owner is this cat*). It also included two randomised attention checks to help identify inattentive participants and unreliable responders (e.g., *how purple is this cat* and *how likely is this cat to turn into a duck*). All questions were answered using a sliding scale, anchored at each end by either ‘not at all [target characteristic]’ versus ‘extremely [target characteristic]’, and one in the middle for ‘moderately [target characteristic]’ For example, for the item ‘*how shy is this cat?*’, the sliding scale had ‘not at all shy’ on the far left, ‘moderately shy’ in the middle, and ‘extremely shy’ on the far right.

### 2.3. Procedure

Ethics approval was obtained from the La Trobe University Human Research Ethics Committee (HEC25196). Information about the study was then disseminated by PetRescue using established public-facing social media channels (e.g., Facebook) and mailing lists. Included in the recruitment materials was the statement that, in appreciation for completing the survey, an AUD 3 donation per participant, up to AUD 2000 total, would be made to Maneki Neko Cat Rescue Inc. (Eaglemont, Victoria, Australia) a cat welfare charity. Interested recipients were invited to take part in a survey hosted on REDCap, an online platform that provides software to create and manage surveys, by clicking on the link provided. This link took them to the Participant Information and Consent form, and after confirming eligibility and consent, they were able to proceed to the survey questions. The entire survey took approximately 30 min to complete, after which participants were presented with a gratitude statement. Data collection proceeded from July to October 2025.

### 2.4. Data Analysis

The data were analysed using SPSS version 29. The dataset was cleaned using listwise deletion, and assumptions were checked following guidelines outlined by Pallant [29]. It was noted while cleaning the data that a much larger number of participants (*n* = 189; 30% of 624) than expected had failed one or two of the attention checks, even though they completed the entire survey. Discussion among the research team led to the belief that this was due to the use of sliding scales, whereby participants were required to shift a cursor from a neutral midpoint to the endpoint of the scale to respond correctly to these items, with even a small deviation from zero being coded as an incorrect response. It was also noted that several participants wrote in free-text responses that they had intentionally answered the attention check questions with a non-zero response because they were perceived as nonsensical. Hence, we decided not to use these questions to exclude participants. Instead, we screened for other indicators of inattention, such as completing the section too quickly, incomplete responses, and straight-lining responses. We did not find any systematic evidence of these indicators.

Descriptive statistics were produced to summarise the data. Relevant assumptions were tested and mostly met. One three-way ANOVA was used to test whether adoptability varied based on coat colour, eye shape, or pupil size. When interaction effects were significant, Tukey’s post hoc tests were used to determine which specific groups differed from each other.

To understand the relationship between adoptability and perceived behavioural traits, we first attempted an exploratory factor analysis to reduce the number of behavioural variables. However, the dataset was unsuitable due to the poor reliability of the resulting subscales. Therefore, we used a stepwise multiple regression to investigate this relationship, because it is suitable for use with many variables in large samples [30]. The regression analysis included adoptability as the dependent variable, and independent variables included all 17 behavioural traits, plus cuteness. Cuteness was included because, even though it is not a behavioural trait, a ’cute’ appearance was expected to have an influence on adoptability [31]. Attractiveness was excluded because it was highly correlated with cuteness (*r* = 0.78, *p* < 0.001), indicating that both variables were effectively measuring the same construct.

Seven two-way ANOVAs were performed to determine whether differences in cuteness or the behavioural ratings identified using the stepwise regression were affected by the manipulated physical traits that significantly predicted adoptability in the three-way ANOVA (i.e., coat colour and eye shape). Tukey post hoc tests were used to investigate relationships among individual variables when the ANOVA results were significant. Due to the multiple analyses, a Bonferroni correction was applied to avoid Type I errors. The overall alpha level for each of these seven ANOVAs was *p* = 0.007.

## 3. Results

### 3.1. Participant Demographics

Table 1 presents demographic information. Participants’ ages ranged from 18 to 86 years (*M* = 50.24, *SD* = 16.25). Of the 624 participants, most were current cat owners, and most identified as female.

### 3.2. Cat Adoptability Ratings Across Experimental Groups

Table 2 presents mean scores on adoptability ratings for each of the 36 cat images. The grey tabby cat with round eyes and small pupils had the highest mean rating of adoptability, but cats with black coats had generally higher adoptability ratings compared to other coat colours. Cats with walnut and round eyes also typically had higher ratings on adoptability, but differences across pupil size were less marked.

A three-way ANOVA comparing how a cat’s coat colour, eye shape, and pupil size impact the cat’s overall adoptability ratings among the thirty-six cat images revealed a significant interaction effect between coat colour and eye shape (*F*_(35, 588)_ = 2.369, *p* = 0.029, η^2^_p_ = 0.024). There was also a significant main effect for coat colour (*F*_(35, 588)_ = 7.818, *p* ≤ 0.001, η^2^_p_ = 0.038). No other main or interaction effects were significant. Post hoc tests revealed a significant difference between cats with black coats and round eyes (*M* = 87.62, *SD* = 23.04) compared to cats with white coats and almond eyes (*M* = 67.85, *SD* = 26.61). Similarly, cats with tabby coats and round eyes (*M* = 86.00, *SD* = 16.07) differed significantly from cats with black coats and walnut eyes (*M* = 85.63, *SD* = 18.43). Overall, there was also a significant difference between cats with black coats (*M* = 80.49, *SD* = 23.24) and cats with white coats (*M* = 67.90, *SD* = 27.53, *p* < 0.001), with black-coated cats obtaining higher means for adoptability.

### 3.3. Characteristics That Predict Cat Adoptability

A stepwise multiple linear regression was used to assess whether cuteness or behavioural characteristics influenced people’s perceptions of a cat’s adoptability. The overall model was significant, *F*_(7, 616)_ = 59.71, *p* ≤ 0.001, and explained 40% of the variance in cat adoptability, R^2^ = 0.404. As presented in Table 3, the characteristics of ‘cute’ (*b* = 0.587, *p* ≤ 0.001), ‘friendly’ (*b* = 0.228, *p* ≤ 0.001), ‘shy’ (*b* = 0.177, *p* ≤ 0.001), ‘active’ (*b* = 0.124, *p* = 0.004), ‘difficult’ (*b* = −0.082, *p* = 0.039), ‘playful’ (*b* = −0.128, *p* = 0.012), and ‘devoted’ (*b* = 0.093, *p* = 0.030) were all statistically significant unique predictors. However, the ß-values were less than 0.10 for the predictors ‘active’, ‘difficult’, ‘playful’, and ‘devoted’, indicating a negligible effect size. Thus, these variables were excluded from further analyses.

### 3.4. Ratings Across Experimental Groups for Significant Predictors of Adoptability Ratings

Because pupil size was not a significant predictor of adoptability in the three-way ANOVA results reported above, it was not included in the following analyses. Instead, two-way ANOVAs were used to determine if images with different coat colours and eye shapes differed on the variables that predicted adoptability in the stepwise regression and had ß-values over 0.10.

A summary of the descriptives for the variables included in the two-way ANOVAs, based on image characteristics, is presented in Table 4.

#### 3.4.1. Cute

A two-way ANOVA, with Bonferroni correction applied, revealed that there were no significant interaction effects. There were, however, significant main effects for coat colour (*F*_(11, 612)_ = 4.146, *p* = 0.006, η^2^_p_ = 0.020) and eye shape (*F*_(11, 612)_ = 10.114, *p* ≤ 0.001, η^2^_p_ = 0.032). Post hoc comparisons using the Tukey HSD test found that cats with black coats were perceived as cuter than those with white coats (*p* = 0.012). Similarly, it was found that cats with round eyes were perceived as cuter than those with almond eye shapes (*p* < 0.001).

#### 3.4.2. Friendly

A two-way ANOVA with Bonferroni correction also revealed that there were no significant interaction effects. There were significant main effects for coat colour (*F*_(11, 612)_ = 8.639, *p* ≤ 0.001, η^2^_p_ = 0.041) and eye shape (*F*_(11, 612)_ = 5.569, *p* = 0.004, η^2^_p_ = 0.018). Post hoc comparisons indicated that cats with black coats were perceived as friendlier than cats with ginger (*p* = 0.001), grey tabby (*p* = 0.027), or white coats (*p* ≤ 0.001). Similarly, cats with round eyes were perceived as friendlier than cats with almond eyes (*p* = 0.006).

#### 3.4.3. Shy

A two-way ANOVA with Bonferroni correction revealed no significant interaction effects or main effects when ‘Shyness’ ratings were compared across groups.

## 4. Discussion

This study aimed to investigate whether ratings of cat adoptability varied with coat colour, eye shape, or pupil size and, if so, whether this may be due to their effects on ratings of cuteness and perceived behavioural traits. Participants shared their perceptions of the overall adoptability of a single cat image, manipulated to show different physical characteristics. Grey tabby cats with round eyes had the highest adoptability ratings, but black cats with all three eye shapes were also rated as highly adoptable. In a regression analysis, seven items explained 40% of the variance in adoptability ratings, with cuteness and the perceived behavioural traits of friendliness and shyness making significant unique predictions. A series of two-way ANOVAs then revealed that ratings of cuteness and friendliness varied with the coat colour and eye shape. No significant interactions were observed. Post hoc tests generally revealed that black cats received more positive ratings than images of cats with different coat colours, while cat images with round eyes were perceived more positively than cat images with almond-shaped eyes.

Although the observed effects were quite modest, it is remarkable that simply changing the coat colour or eye shape of a cat image can significantly influence ratings of adoptability, as well as ratings of traits like cuteness, friendliness, and shyness. To our knowledge, this has not previously been demonstrated. Moreover, the finding that black cats were rated as most adoptable overall contradicts some previous research, which has concluded that lighter-coloured cats are typically adopted sooner, presumably because they are perceived to display more positive behavioural traits [11,28]. Participants in the current study rated black cats as being friendlier than other coat colours. It is unclear why this is the case, but it does accord with a previous study in Sydney, Australia, showing that white cats stayed longer in a shelter than black cats [12].

Much of the existing research on cat colour and perceptions of friendliness and adoptability is based in the USA [14,15,32], which is culturally similar to Australia, so cultural differences are unlikely to explain the disparity. On the other hand, Australian shelters sometimes put indemnity waivers on cats available for adoption if they have been diagnosed with, or are at risk of, certain diseases [33]. This transfers financial and healthcare responsibilities to the adopter. One of these waivers relates to skin cancer and is used in cats with pale fur/skin. A 2019 study found that the presence of indemnity waivers was related to an increased length of stay in the shelter [34]. It is therefore possible that our participants were aware of this practice and had a positive bias towards black cats due to a reduced likelihood of skin cancer.

Perhaps this specific population, recruited through PetRescue, a national animal welfare charity, was educated about ‘black dog syndrome’ and ‘black cat bias’ (i.e., the perception that black dogs and cats are less likely to be adopted than other colours). Indeed, PetRescue has regular communications with its followers about black cat adoption [20,34]. Furthermore, we did not collect data on whether participants were explicitly involved in animal rescue (e.g., foster carers, shelter staff), but it is possible that some of them were. PetRescue is not an adoption organisation, per se, but rather an adoption advertising website used by approximately 800 individual adoption organisations. Recruitment proceeded via PetRescue’s public-facing channels (e.g., Facebook: 207,000 followers; Instagram: nearly 60,000 followers; public mailing list: over 250,000 subscribers), rather than their member-facing channels, so we anticipate that most of the participants are likely members of the general public, rather than those involved in adoption.

In the recruitment ad, there was no mention of coat colour, but it was mentioned in the information statement that participants read before beginning the survey, so they would have been aware that coat colour was being investigated. Nonetheless, the empirical evidence for black cat bias is not straightforward [14,21]. For instance, one study using an implicit association test found that black cat bias was present in dog people and cat and dog people, but not in cat people [15]. In the same study, belief in witchcraft was a predictor of black cat bias, but not general superstitiousness [15]. A different study, however, found that superstitiousness was associated with black cat bias, as well as difficulty reading the emotions of black cats [14]. It is unclear to what extent black cat bias truly affects adoption outcomes for shelter cats.

Although eye shape on its own was not a predictor of adoptability, there was an interaction effect between coat colour and eye shape. Generally, cats with rounder eye shapes received higher ratings for adoptability. This may relate to the perceived cuteness of the cat, which was also higher for cats with round eye shapes. Previous literature has found that features of the eye region of any species are extremely important when influencing perceptions of cuteness [3], with baby schema theory explaining this well-established phenomenon [35]. Furthermore, the results of eye shape influencing cuteness concurred with research by Jack and Carroll [6], which found that individuals perceived edited images of cats with rounder eyes as cuter.

Pupil size did not lead to significant differences in ratings for adoptability. This is surprising as previous research suggests that dilated pupils are evaluated more positively [36], but it is possible that the images used in the current study were too small for pupil size to be easily perceived. While we used a published sizing guide for cat pupils [24] to ensure the three sizes were easily distinguishable in life-size images, it is likely that many participants completed the survey using small devices that obscured these differences. We did not specifically check for this in our piloting.

These results support previous research that potential adopters choose cats based on their perceived cuteness, as well as behavioural traits associated with sociability [3,37,38] and expand on this previous research by confirming that these traits are influenced by physical features depicted in a cat image, where all other contextual features were held constant. Perceived cuteness is believed to indicate vulnerability, leading to adoption in order to nurture and care for the cat [16,39]. This is consistent with the theoretical perspective, which holds that the baby schema is very influential in multiple contexts. Features of baby schema morphology, specifically round eyes, led to a noticeable increase in the cat’s cuteness ratings, as well as the cat’s adoptability ratings when combined with black coats, possibly by awakening an instinctual sense of nurturance [35,40]. Behavioural traits, particularly those associated with sociability (e.g., friendly, playful, not difficult), are also well established as being highly desirable amongst cat adopters [3]. Sociability is also an important factor for a cat owner’s satisfaction with their cat [41].

This research can potentially help animal adoption agencies improve how they present cats available for adoption online. These organisations can use the information to understand which features of cats, including the cats’ coat colour and eye shape, need to be highlighted in profile images. They may also be able to counteract any negative impacts of specific physical traits by explicitly drawing attention to desirable behavioural traits, perhaps by adding contextual features such as toys or other animals. Such strategies could increase the likelihood of adoptability for available cats, particularly those with less desirable physical features. Cuteness is particularly important, so including elements in the images of cats to help them appear cuter to potential adopters may increase the likelihood of adoptability. Furthermore, the most important behavioural characteristics for a cat to display appear to be those that align with sociability. It seems that even superficial changes to a cat’s appearance lead potential adopters to make relatively consistent inferences about behavioural features that simply cannot be reliably deduced from a static image. Therefore, emphasising a cat’s friendliness through text accompanying the images could be critical for enhancing perceived adoptability.

Testing this was beyond the scope of this project, but further research is recommended to assess the effectiveness of explicitly advertising these qualities in text, particularly when the physical features of the cat are less likely to result in a positive outcome. It would also be of value to repeat the study in a wider sample from different backgrounds. There were many more females than males in our sample. This limits the generalizability of the results but is a problem common in human–animal relationship research [42]. It is also in line with the gender breakdown of PetRescue’s social media following, which is approximately 85% women on both Facebook and Instagram. In addition, we intentionally recruited through PetRescue so that the sample would consist mostly of people with an interest in cat adoption. This also increased the likelihood that this specific population may have had pre-existing knowledge on cat adoption trends, which may have influenced how they perceived each cat and, therefore, potentially affected the results.

Even varying the type of cat might influence the results obtained. We chose to use a domestic shorthair cat as these are the most common cats available for adoption, but the results may not apply to breeds with more distinctive facial or coat characteristics, or cats with other coat colours or eye shapes than those we utilised. Further research should continue to investigate how various physical features interact to influence adoptability ratings and whether physical features interact with contextual features (e.g., toys and natural settings) or accompanying text to determine perceptions of adoptability, cuteness, and behaviour. Any strategy to increase adoption rates has the potential to improve animal welfare outcomes for shelter cats [43]. Hence, future research examining these issues could help shelters and adoption organisations develop nuanced strategies to facilitate finding homes for cats in need.

## 5. Conclusions

This study suggests that a cat’s adoptability may be related to perceptions of the cat’s cuteness and specific behavioural traits, which, in turn, may vary according to the cat’s coat colour and eye shape. Black cats were generally ranked as more adoptable than other colours. Information about how features such as coat colour, eye shape, and pupil size can influence adoptability is important for organisations seeking to find homes for cats, potentially helping them structure images and other features of online advertisements in ways that make individual cats more appealing to potential adopters.

## Figures and Tables

**Table 1:** Sample demographics. Due to small amounts of missing data, 623 participants completed their age, and 622 participants completed their gender and cat ownership status.

Demographics	*n*	%
**Gender**		
Male	48	7.7
Female	552	88.5
Non-binary/third gender/self-described/rather not say	22	3.5
**Age**		
18–24	35	5.6
25–34	94	15.1
35–44	109	17.5
45–54	124	19.9
55–64	118	18.9
65 and over	143	22.9
**Cat Owner**		
Current owner of a cat	513	82.2
Not a current owner of a cat	109	17.5

**Table 2:** Descriptive statistics of adoptability ratings for each of the 36 photos, in descending order of mean score.

Photo Characteristics	*n*	Mean	Median	Standard Deviation	Observed Range
Tabby, round, small	13	91.00	99.00	15.17	50–100
Black, walnut, large	9	89.44	97.00	16.24	50–100
Black, walnut, medium	18	85.06	94.00	18.85	50–100
Black, round, medium	12	84.67	92.00	17.88	50–100
Black, walnut, small	13	84.15	81.00	17.10	50–100
Black, almond, small	14	81.50	85.50	18.94	50–100
Tabby, round, large	20	80.95	85.00	20.17	39–100
Ginger, walnut, small	27	80.81	87.00	23.47	6–100
Black, almond, medium	17	80.29	80.00	22.72	27–100
Tabby, almond, small	18	79.56	79.00	19.81	50–100
Ginger, almond, medium	15	79.47	80.00	20.52	38–100
Black, round, small	11	79.45	99.00	31.84	0–100
Tabby, almond, medium	19	79.32	80.00	16.41	50–100
Tabby, round, medium	16	77.31	86.00	25.49	15–100
Black, round, large	19	77.00	100.00	29.76	14–100
Ginger, walnut, medium	22	76.82	82.00	24.05	29–100
White, round, medium	15	76.60	88.00	23.97	41–100
Ginger, walnut, large	20	75.30	75.50	21.41	35–100
Ginger, round, small	17	75.24	79.00	22.45	31–100
Tabby, walnut, medium	25	74.36	80.00	21.99	29–100
Tabby, walnut, large	19	73.53	75.00	19.86	37–100
Black, almond, large	24	72.42	80.00	26.35	20–100
White, walnut, large	16	72.06	87.50	29.29	20–100
White, walnut, medium	17	71.35	82.00	32.34	8–100
Ginger, almond, small	17	71.24	77.00	24.70	28–100
Ginger, round, large	15	71.20	75.00	25.86	25–100
White, almond, medium	18	70.72	75.50	24.44	8–100
Tabby, almond, large	19	69.95	71.00	22.57	25–100
Ginger, almond, large	22	68.82	75.50	25.78	15–100
White, walnut, small	16	67.63	70.00	26.12	22–100
White, round, small	22	67.59	65.50	28.16	20–100
White, round, large	12	67.08	68.00	17.15	50–10
Tabby, walnut, small	20	63.65	57.50	28.77	12–100
Ginger, round, medium	18	62.11	55.50	28.20	0–100
White, almond, small	15	59.53	55.00	30.99	6–100
White, almond, large	14	56.43	57.00	31.73	6–100

**Table 3:** Stepwise multiple linear regression of characteristics predicting cat adoptability ratings. Four predictors, active, difficult, playful, and devoted, were excluded due to negligible ß-values, even though they were statistically significant.

Characteristics	*b*	*SE*	ß	*t*	*p*
Cute	0.587	0.039	0.514	15.021	**<0.001**
Friendly	0.228	0.055	0.171	4.137	**<0.001**
Shy	0.177	0.042	0.135	4.186	**<0.001**
**Excluded Variables**	***b***	***SE***	**ß**	***t***	***p***
Active	0.124	0.043	0.098	2.879	**0.004**
Difficult	−0.082	0.039	−0.070	−2.072	**0.039**
Playful	−0.128	0.051	−0.099	−2.522	**0.012**
Devoted	0.093	0.043	0.078	2.178	**0.030**
Aggressive	−0.045	−0.050	0.721	−1.240	0.215
Aloof	−0.053	−0.064	0.876	−1.595	0.111
Bold	−0.051	−0.063	0.886	−1.558	0.120
Calm	0.037	0.043	0.809	1.059	0.290
Fearful	0.007	0.008	0.716	0.198	0.843
Intolerant	−0.030	−0.035	0.815	−0.878	0.380
Stubborn	−0.053	−0.066	0.906	−1.637	0.102
Tolerant	0.044	0.048	0.708	1.204	0.229
Trainable	0.011	0.013	0.788	0.325	0.745
Shows amicable behaviour towards other cats	0.000	0.000	0.771	−0.011	0.991
Shows amicable behaviour towards young children	0.012	0.013	0.763	0.329	0.743

*Note.* Items highlighted in **bold** are significant at *p* < 0.05.

**Table 4:** Means and Standard deviations for the seven variables included in the two-way ANOVAs, based on cat coat colour and eye shape.

Cat Characteristic(s)	Cute *M (SD)*	Friendly *M (SD)*	Shy *M (SD)*
**Coat colour totals**			
Black Total	82.55 (20.28)	71.36 (19.15)	37.71 (18.99)
Ginger Total	77.08 (21.28)	63.72 (15.79)	34.53 (17.16)
Tabby Total	78.55 (20.18)	65.63 (18.18)	37.98 (18.55)
White Total	74.81 (23.99)	61.32 (20.10)	38.59 (20.48)
**Eye shape totals**			
Almond Total	73.78 (22.84)	62.56 (19.86)	37.65 (19.94)
Round Total	82.79 (19.30)	68.09 (18.41)	35.95 (18.41)
Walnut Total	78.35 (21.83)	65.62 (17.00)	37.58 (17.95)
**Interactions**			
Black + Almond	76.44 (23.04)	68.35 (20.88)	38.15 (19.24)
Black + Round	87.62 (16.02)	75.43 (18.58)	35.90 (17.77)
Black + Walnut	85.63 (18.43)	71.25 (16.78)	39.00 (20.17)
Ginger + Almond	72.65 (20.52)	58.67 (15.24)	35.67 (18.68)
Ginger + Round	79.08 (21.01)	64.10 (16.50)	31.24 (15.47)
Ginger + Walnut	79.10 (21.84)	67.39 (14.82)	36.03 (17.01)
Tabby + Almond	77.25 (20.77)	63.95 (16.93)	37.71 (17.67)
Tabby + Round	86.00 (16.07)	71.29 (16.01)	38.27 (22.17)
Tabby + Walnut	73.98 (21.13)	62.52 (19.97)	38.00 (16.44)
White + Almond	67.85 (26.61)	58.60 (24.70)	39.26 (24.61)
White + Round	79.24 (21.91)	62.67 (20.05)	38.49 (17.14)
White + Walnut	77.04 (22.26)	62.59 (14.65)	38.04 (19.59)

## Data Availability

Due to ethical restrictions, the data can only be made available upon request to the authors and approval from the relevant Ethics Committee.

## Supplementary Materials

The following supporting information can be downloaded at: https://www.mdpi.com/article/10.3390/ani16020339/s1, Material S1: Image Combinations of Variables; Material S2: Prompts for AI 4o Image Generation.
