# Supplementary material for: The Influence of Cat Coat Colour, Eye Shape, and Pupil Size on Ratings of Adoptability Based on a Standardised Online Image, in an Australian Sample
Source: Animals (Basel). 2026 Jan 22;16(2):339. doi: 10.3390/ani16020339 (PMC12837537; doi:10.3390/ani16020339)

Supplementary Material S1: Image Combinations of Variables

Round Eye Shape

Pupil Size: Large = L, Medium = M, Small = S

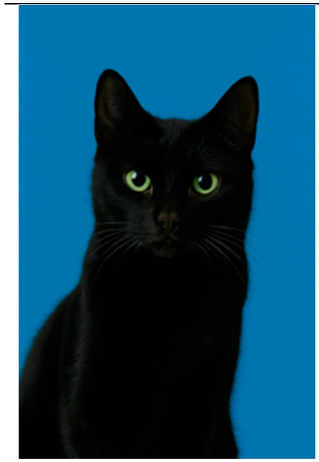
L

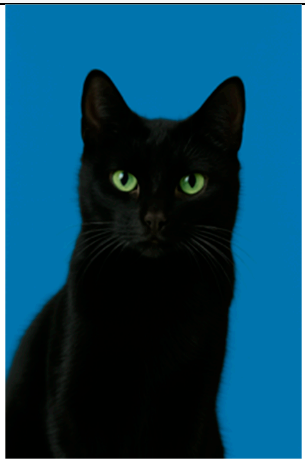
M

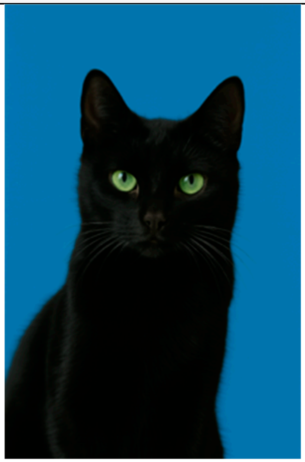
S

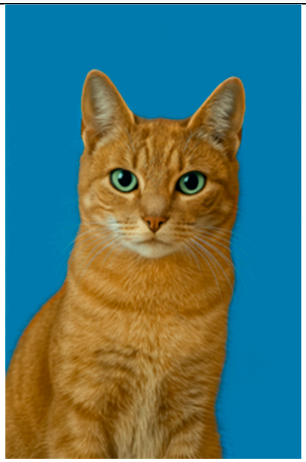
L

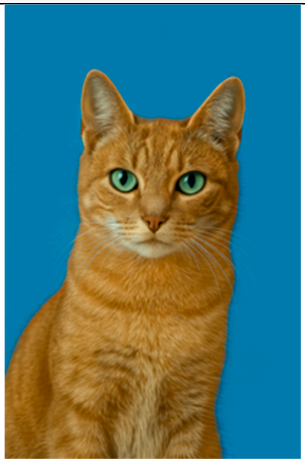
M

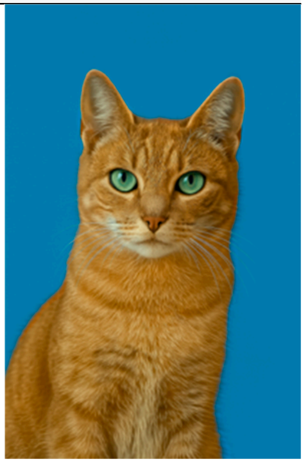
S

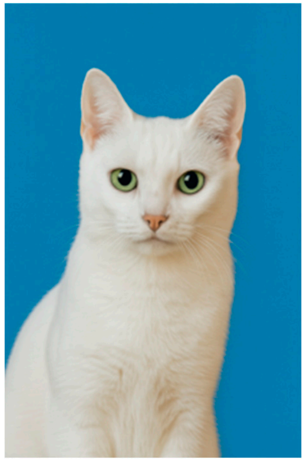
L

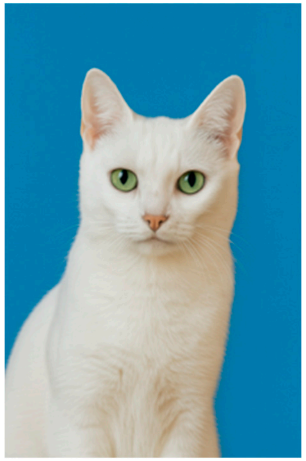
M

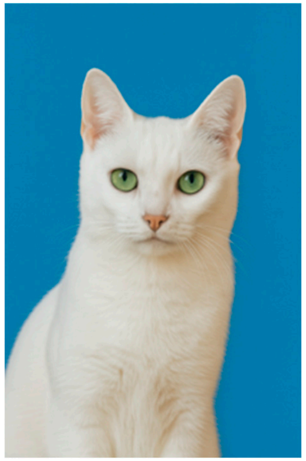
S

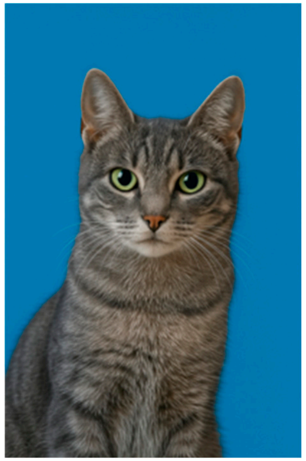
L

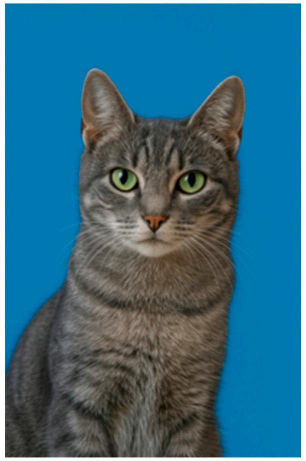
M

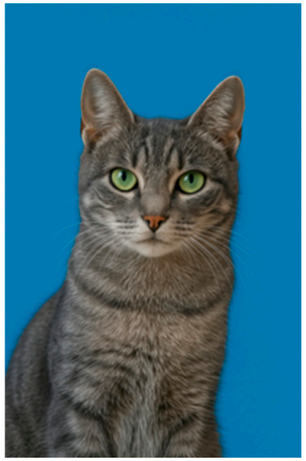
S

Almond Eye Shape

Pupil Size: Large = L, Medium = M, Small = S

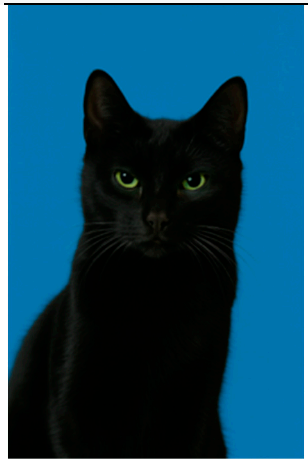	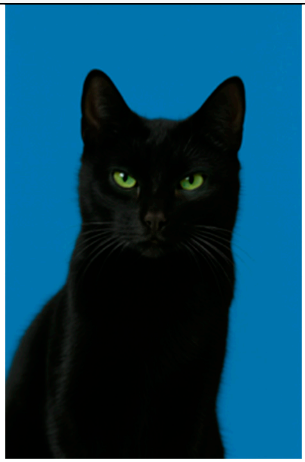	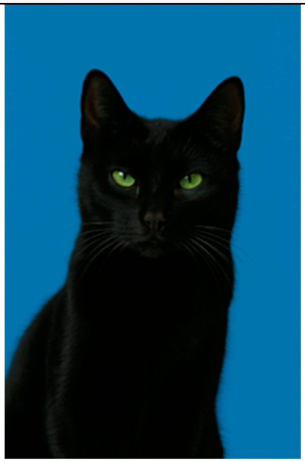	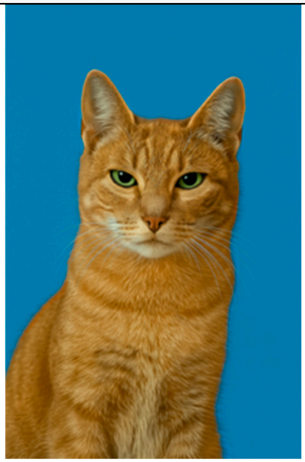	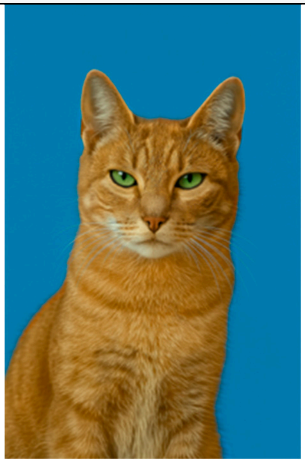	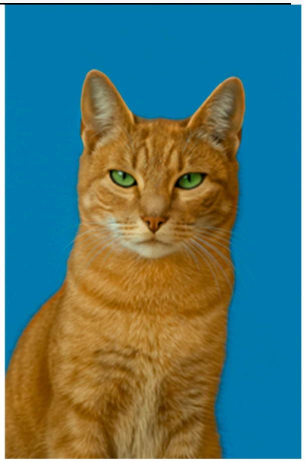
L	M	S	L	M	S
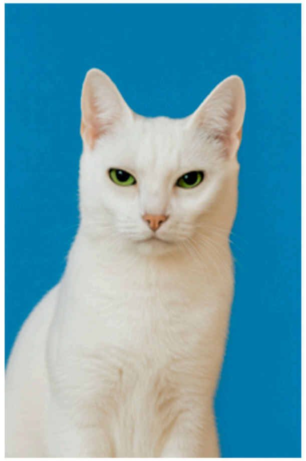	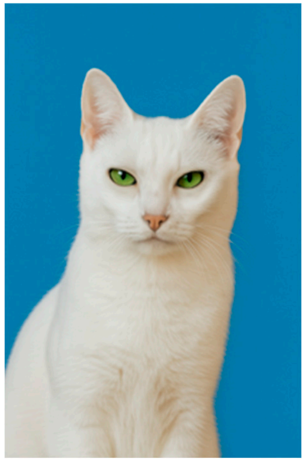	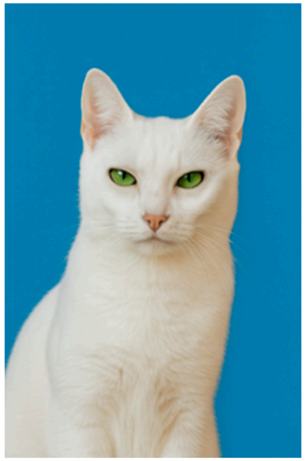	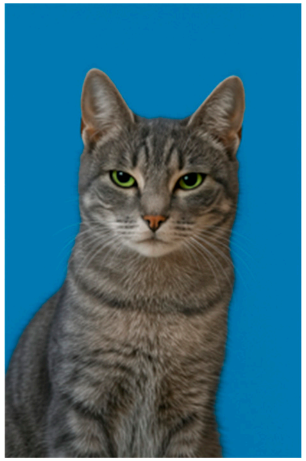	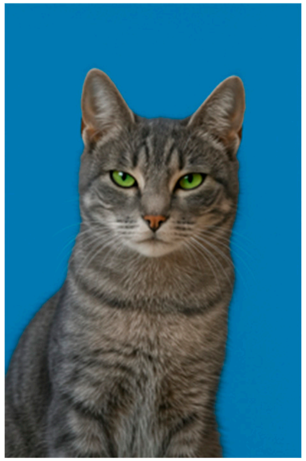	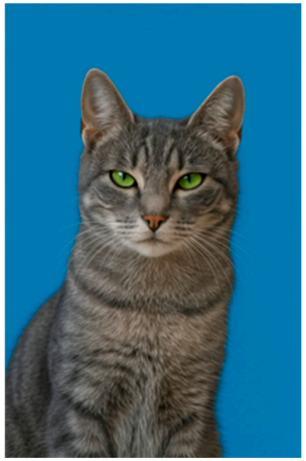
L	M	S	L	M	S

Walnut Eye Shape

Pupil Size: Large = L, Medium = M, Small = S

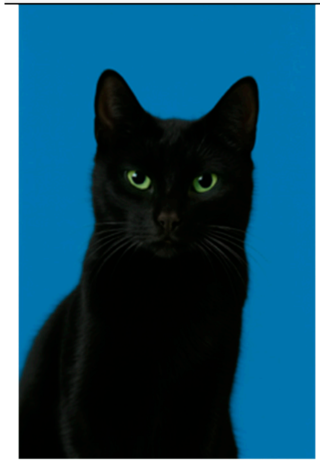	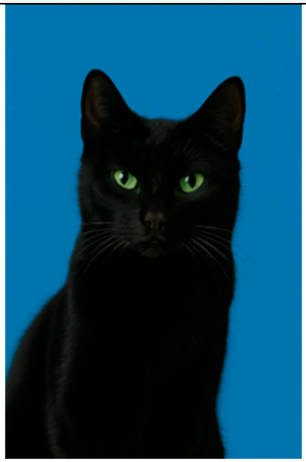	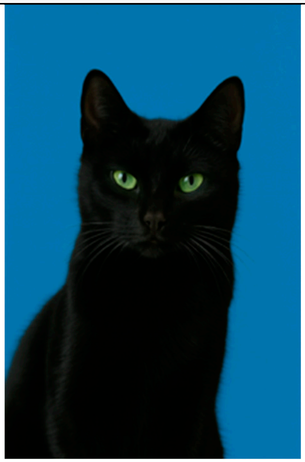	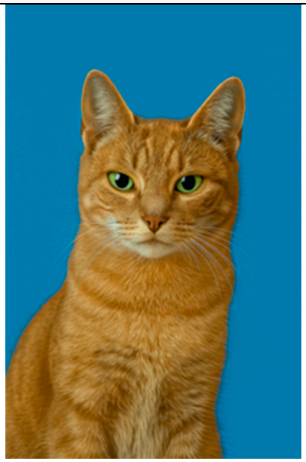	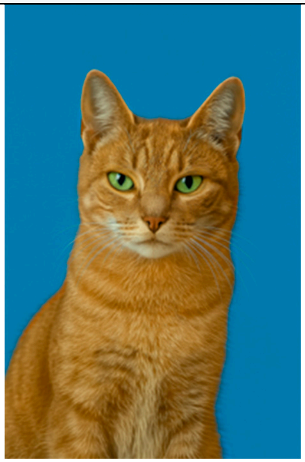	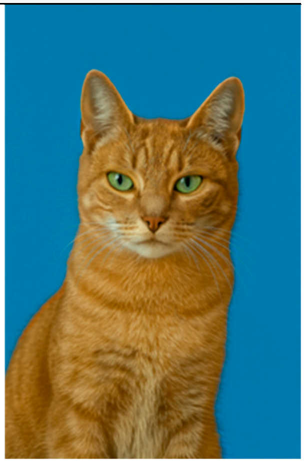
L	M	S	L	M	S
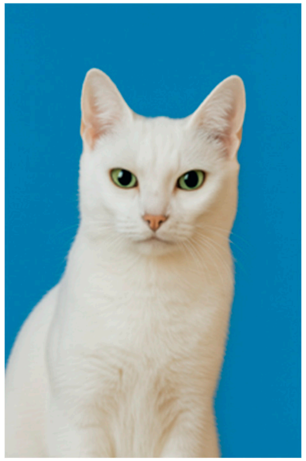	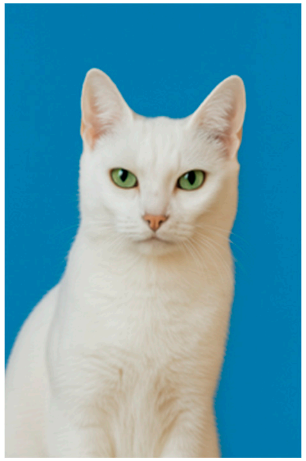	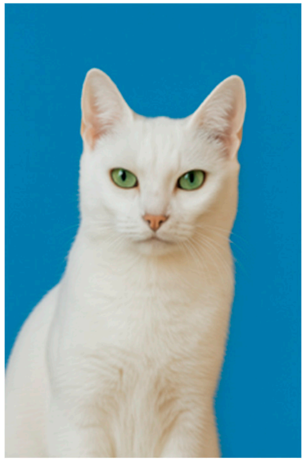	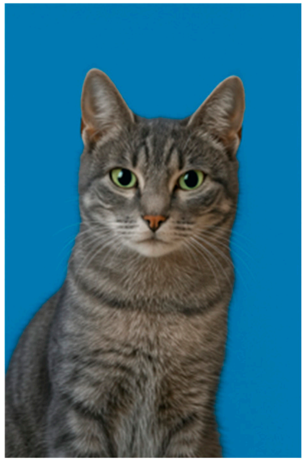	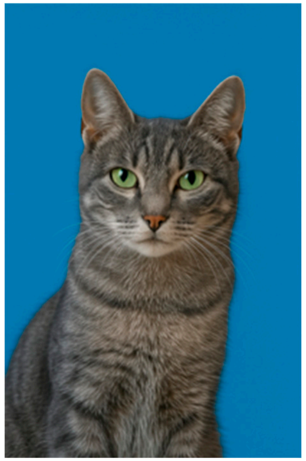	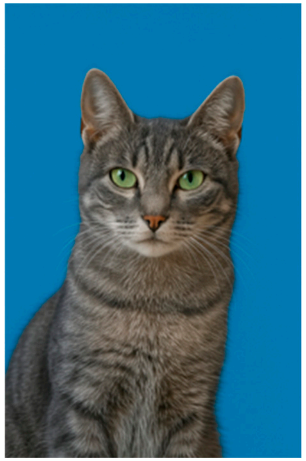
L	M	S	L	M	S

Supplementary Material S2: Prompts for AI 4o Image Generation

Prompt 1: Setting the context

Hi! I have a brief for some image editing for a research experiment about cat attractiveness and would like your help in generating images for me for this study with a focus on preserving the required aspects without changes to the original photo and only editing one variable at a time (the eyes, which I will share example images of). Are you ready for the brief? How would it be best to provide you the information to ensure that the image will be as requested?

Prompt 2:

This is exactly what is needed:

1. The background for the AI cat photos should be a solid blue colour. As research has depicted that a solid background helps to keep focus on the pets, while blue allows for great contrast between the four coat colours that we are investigating.
2. All cat images need to be the same for consistency, so we need to recreate the original even though we want it to be the same colour
3. We are seeking 7 images to be edited and provided depicting the original cat on the new background as the default primary image, and then edited to depict the 3 eye shapes, noting that the eye colour for all the cats in the AI image will be green as per the colour in this original image. NOTE for emphasis: the rest of the image should remain unchanged. Only the eyes need the editing for the purpose of this experiment.

Are you ready for the images, and should we do this step by step so that I can verify you are meeting the brief along the way? Do you need any more information to avoid assumptions?

<u>Prompt 3</u>:

Here is the image of the cat. Let's start with this and then we move onto the eye shape variation examples (3).

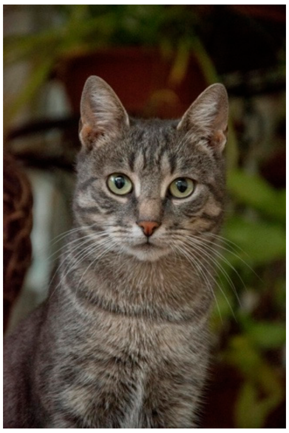

From *Close-Up Shot of a Brown Tabby Cat*, by B. Claudia, 2018, Pexels (https://www.pexels.com/photo/close-up-shot-of-a-brown-tabby-cat-10589798/). CC BY 3.0

<u>Prompt 4</u>:

Take this original photograph of a grey tabby cat and only replace the background with a solid flat colour: #4A90E2. Do not regenerate a new version of the cat, use the image editing tool to achieve the background change. Do not alter the cat in any way — this includes: - No changes to fur tone or texture - No changes to the eye colour or reflections - No smoothing, filtering, or enhancements - No cropping, resizing, or re-centring - The output should be the exact same cat photo, with the only difference being the background colour.

<u>Prompt 5</u>:

Edit this exact photo of a grey tabby cat so that only two things are changed:

1. Replace the background with a solid colour: #4A90E2 (flat blue).

2. The colour temperature of the cat's fur should be cool - if necessary, reduce warmth and make the coat appear more neutral grey, so that the tone is exactly as per in the original photo.

Important editing constraints:

- Do not regenerate or change the shape, structure, or pose of the cat.
- Maintain all original textures, facial features, fur patterns, and lighting.
- Do not alter eye shape, colour, reflection, or sharpness.
- Do not apply smoothing or filters — keep detail sharp and natural.
- Do not reframe or crop — preserve the exact original photo proportions and position.

The result for Image 1 should be the same cat photo, only with a cooler-toned grey coat and solid blue background. Call this the base image for future referencing.

Results of prompt 5:

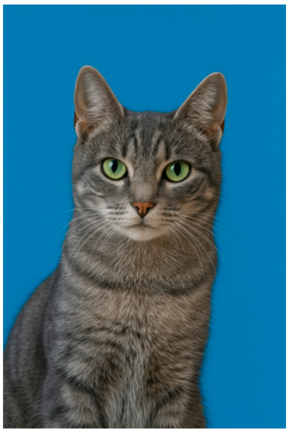

Prompt 6:

Please take this base image and provide me with the following image variations with strict rules applied. Change the fur colour tone and hues ONLY without editing or manipulating the rest of the image.

1. The colour to change to is Ginger tabby (but please ensure that the colour is a realistic tone, VERY realistic – not too bright or Fluro!)
2. The colour to change to is: White (but please ensure that the colour is a realistic tone, VERY realistic – not too bright or Fluro!)
3. The colour to change to is: Black (but please ensure that the colour is a realistic tone, VERY realistic – not too bright or blur softened or Fluro!)

The rest of the image - including face shape, eyes, fur texture and print details, lighting, background, facial features, orientation, and expression - must remain completely unchanged. Do not reframe, regenerate, or alter the structure of the cat's head or body. Eyes must remain the same, realistic and consistent with feline anatomy. Do not change the colour of the eyes from the original.

Results of prompt 6:

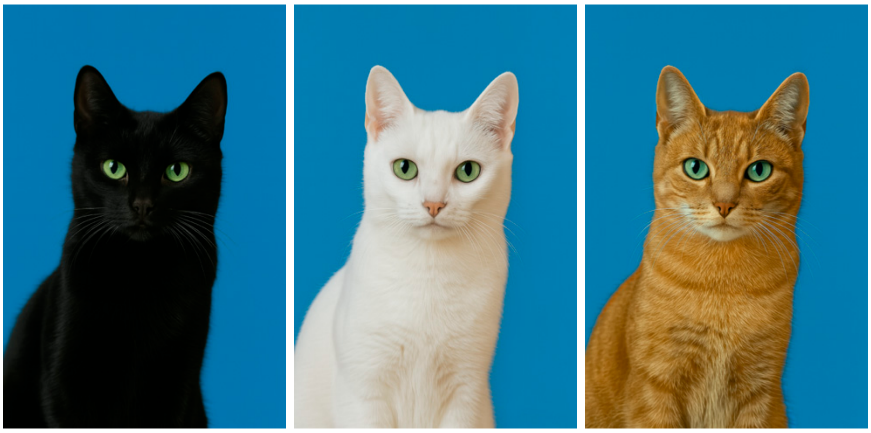

Prompt 7:

Please take this base image and provide me with the following image variations with strict rules applied.

Edit only the eyes to create walnut-shaped eyes with these properties: A blend of almond and round — horizontally wider than tall, but not circular, realistic in proportion to face. Outer corners slant upward slightly. Lower eyelid should appear flatter than the round shape. Upper

eyelid should arch gently, not steeply. The overall shape should resemble that seen in Abyssinian or Burmese cats. Do not change the colour of the eyes. Mood: Observant, intelligent, serene. DO NOT EDIT OR CHANGE ANYTHING ELSE IN THE IMAGE asides from the coat colour and the eye shape as described above.

Results of prompt 7:

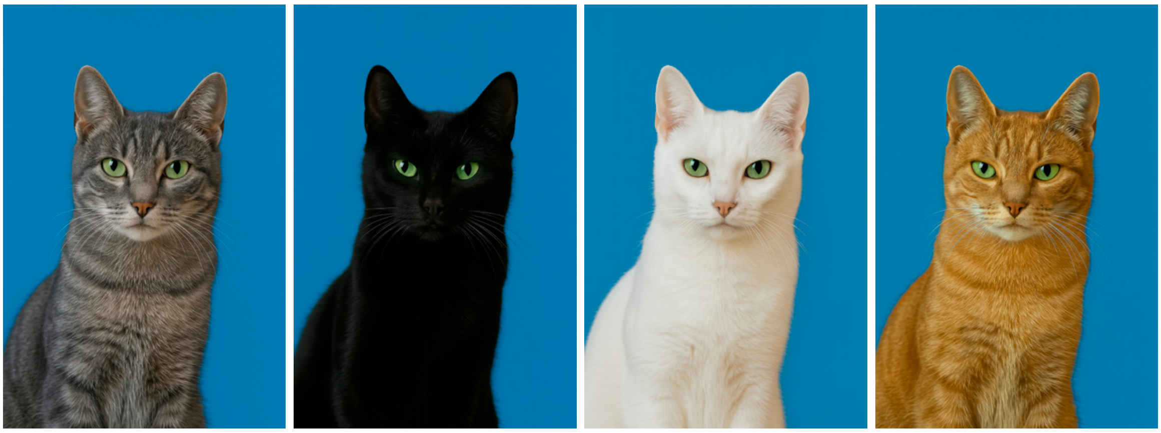

Prompt 8:

Please take this base image and provide me with the following image variations with strict rules applied.

Edit only the eyes to create walnut-shaped eyes with these properties: A blend of almond and round — horizontally wider than tall, but not circular, realistic in proportion to face. Outer corners slant upward slightly. Lower eyelid should appear flatter than the round shape. Upper eyelid should arch gently, not steeply. The overall shape should resemble that seen in Abyssinian or Burmese cats. Do not change the colour of the eyes. Mood: Observant, intelligent, serene. DO NOT EDIT OR CHANGE ANYTHING ELSE IN THE IMAGE asides from the coat colour and the eye shape as described above.

Results of prompt 8:

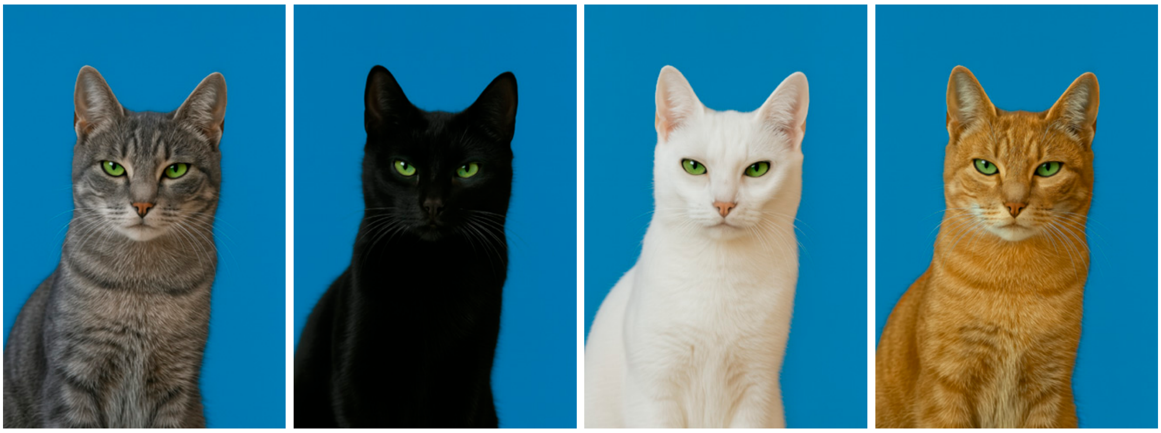

Supplement: Supplementary file 1 [file animals-16-00339-s001.zip › animals-4066569-supplementary.pdf]
